# Cardiac fibrosis in mouse expressing DsRed tetramers involves chronic autophagy and proteasome degradation insufficiency

**DOI:** 10.18632/oncotarget.11026

**Published:** 2016-08-02

**Authors:** Tsung-Hsien Chen, Mei-Ru Chen, Tzu-Yin Chen, Tzu-Chin Wu, Shan-Wen Liu, Ching-Han Hsu, Gan-Guang Liou, Yu-Ying Kao, Guo-Chung Dong, Pao-Hsien Chu, Jiunn-Wang Liao, Kurt Ming-Chao Lin

**Affiliations:** ^1^ Institute of Biomedical Engineering and Nanomedicine, National Health Research Institutes, Zhunan, Miaoli, Taiwan; ^2^ Institute of Biomedical Engineering and Environmental Sciences, National Tsing Hua University, Hsinchu, Taiwan; ^3^ Institute of Molecular and Genomic Medicine, National Health Research Institutes, Zhunan, Miaoli, Taiwan; ^4^ Department of Biotechnology, Chia Nan University of Pharmacy and Science, Tainan, Taiwan; ^5^ Department of Cardiology, Chang Gung Memorial Hospital, Chang Gung University College of Medicine, Taoyuan, Taiwan; ^6^ Graduate Institute of Veterinary Pathobiology, National Chung Hsing University, Taichung, Taiwan

**Keywords:** protein aggregation, cardiac hypertrophy, fibrosis, heart failure, proteasome, Pathology Section

## Abstract

Proteinopathy in the heart which often manifests excessive misfolded/aggregated proteins in cardiac myocytes can result in severe fibrosis and heart failure. Here we developed a mouse model, which transgenically express tetrameric DsRed, a red fluorescent protein (RFP), in an attempt to mimic the pathological mechanisms ofcardiac fibrosis. Whilst DsRed is expressed and forms aggregation in most mouse organs, certain pathological defects are specifically recapitulated in cardiac muscle cells including mitochondria damages, aggresome-like residual bodies, excessive ubiquitinated proteins, and the induction of autophagy. The proteinopathy and cellular injuries caused by DsRed aggregates may be due to impaired or overburdened ubiquitin-proteasome system and autophagy-lysosome systems. We further identified that DsRed can be ubiquitinated and associated with MuRF1, a muscle-specific E3 ligase. Concomitantly, an activation of NF-κB signaling and a strong TIMP1 induction were noted, suggesting that RFP-induced fibrosis was augmented by a skewed balance between TIMP1 and MMPs. Taken together, our study highlights the molecular consequences of uncontrolled protein aggregation leading to congestive heart failure, and provides novel insights into fibrosis formation that can be exploited for improved therapy.

## INTRODUCTION

Cardiac myocytes are terminally differentiated cells with limited capacity of regeneration, therefore particularly sensitive to any stress with the potential of myocyte loss. Excessive misfolded proteins or aggregates in cardiac myocytes are often discovered at late stages of common heart diseases including cardiac amyloidosis, pathologic hypertrophy and dilated/ischemic cardiomyopathies [1–5]. Defects in key components of protein quality control (PQC) systems and high expression of aggregation-prone proteins may overwhelm protein clearance systems, leading to further aggregations that elicit cellular stress and inflammation, myocyte deaths, and ultimately heart failure [6–8]. The deposits of fibrotic extracellular matrix (ECM) are induced by local immune cell activation and the process is tightly regulated by the balanced action of matrix metalloproteinases (MMPs) and tissue inhibitors of metalloproteinases (TIMPs) [9–13]. Although it is widely accepted that proteinopathy plays a significant role in the pathogenesis of heart failure, the mechanisms by which protein aggregates cause injuries in cardiac myocytes and induce interstitial fibrosis are still unclear.

Tetrameric DsRed, a mammalian derivative of red fluorescent protein (RFP) of the coral *Discosoma,* was generated originally for imaging applications, but later it was found toxic to cells when expressed in large amount and the underlying mechanisms were not extensively explored [14, 15]. Tg mouse expressing tetrameric DsRed was not previously feasible presumably due to the toxicity to embryonic stem cells [14, 15]. Here we report the Tg mouse that switches from expressing enhanced green fluorescent protein (EGFP) to expressing DsRed tetramers. Severe cardiovascular phenotypes were resulted in DsRed mice including lethal dilated heart failure and massive fibrosis at 5 months of age. Thus, we hypothesized that the aggregation of DsRed tetramers causes proteinopathy in cardiac myocytes that can potentially overwhelm normal PQC systems and result into hypertrophy and fibrosis. To define the abnormalities in protein clearance pathways, we investigated autophagosome lysosome system (ALS) and ubiquitin proteasome system (UPS) in affected hearts and studied the fibrosis pathways underlying the disease progression. Unlike models of proteinopathy containing a congenital defect of PQC components, DsRed mouse preserves intact PQC system, thus potentially suitable for studies aiming to elucidate mechanistic connection among protein clearance systems, proteinopathy, and cardiac fibrosis.

## RESULTS

### Tetrameric RFP expression results in heart failure in mice

**Figure 1:**
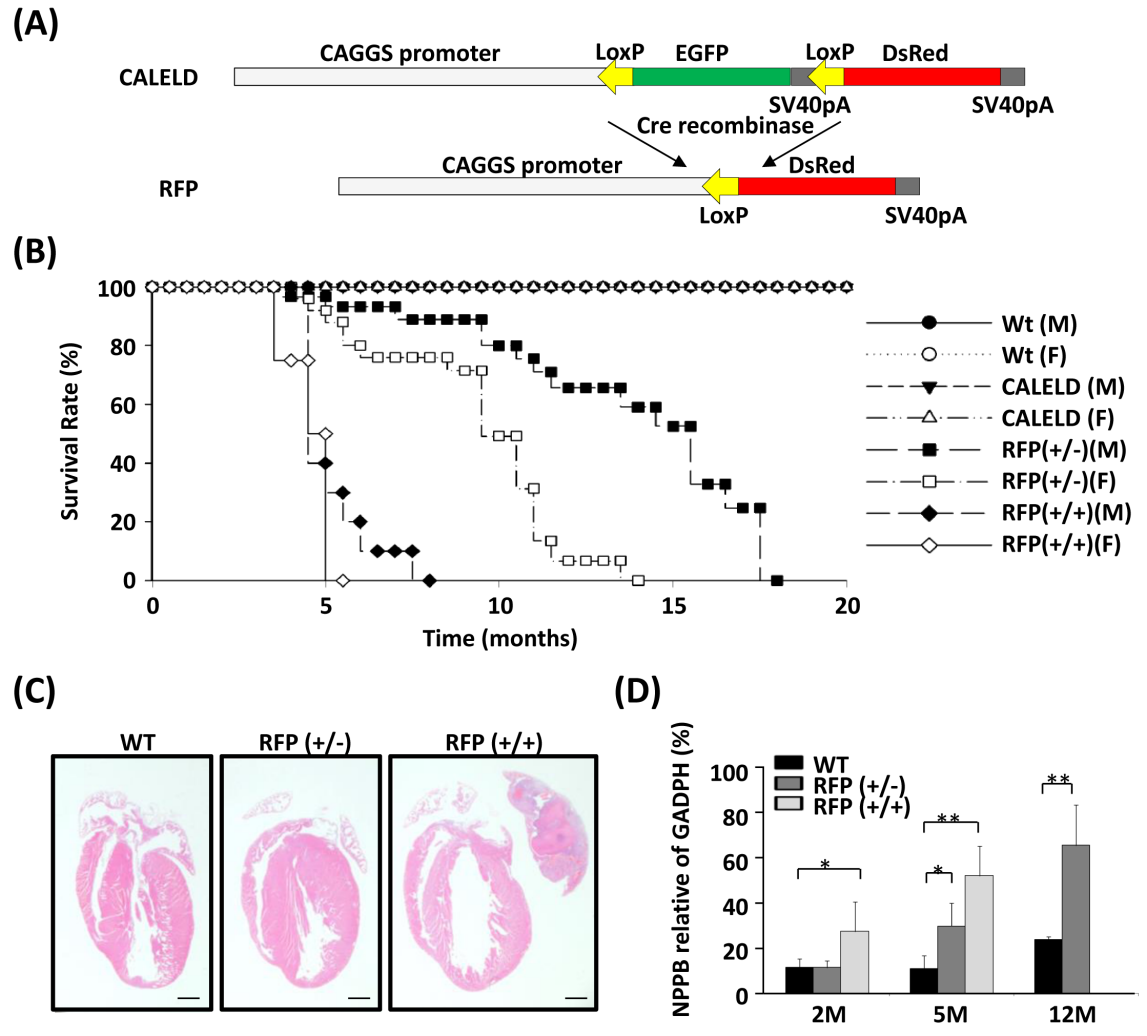
Heart failure in RFP Tg mice **A.** Tg vector for generating conditional CALELD Tg mouse. Tetrameric DsRed cDNA was placed downstream of a LoxP-flanked cassette containing EGFP cDNA, three stop codons and the SV40pA sequence. The CAGGS promoter, a hybrid chicken β-actin and cytomegalovirus promoter, was used to drive the transgene. Following Cre-mediated excision of the EGFP cassette, the tetrameric DsRed cDNA was expressed due to located immediately downstream to the CAGGS promoter. SV40pA, SV40 polyadenylation sequence. **B.** A Kaplan-Meier curve shows the survival rates of male (M) and female (F) Tg mice. The number of mice in comparison: wild-type (Wt) mice (M, 83; F, 84), CALELD(+/+) mice (M, 40; F, 44), heterozygous RFP (+/−) mice (M, 64; F, 69) and homozygous RFP (+/+) mice (M, 82; F, 88). **C.** H&E stained longitudinal sections of hearts from 5-month-old WT, CALELD (+/+) and RFP (+/+) mice. Scale bar = 1 mm. **D.** Expression of NPPB in LV of 2-, 5- and 12-month-old WT and RFP Tg mice. Data shown are normalized to GADPH. **, *P* < 0.01; *, *P* < 0.05 compared with the WT mouse, *n* = 5 for each mouse group.

We previously generated a Tg mouse model, in which the expression of EGFP coding sequence within a LoxP cassette was driven by a ubiquitous CAGGS promoter (Figure 1A). By crossing with a CAGGS-Cre mouse, the green CALELD mouse became a red mouse due to the expression of tetrameric DsRed protein (RFP) (Figure S1A). The RFP mice which do not carry Cre transgene were bred further to generate RFP homozygotes (RFP+/+), which can be distinguished from hemizygotes (RFP+/−) by RT-PCR (Figure S1B) and by visual inspection on red fluorescence of mouse skin or eyes. RFP+/+ or RFP+/− mice were not different from wild-type mice in development and breeding (Figure S1C). Although appearing normal initially, RFP+/+ mice started to develop respiration distress after 4 months of age. Female RFP+/+ mice died at 5 months on average, and the males died at around 7 months. RFP+/− mice have longer life spans, on average 12 months for females and 18 months for males (Figure 1B). After signs of labored breathing appear in moribund RFP mice, cardiac hypertrophy, large left atrium blockage and thrombosis, and lung edema were always observed in the autopsy of euthanized mice (Figure 1C, 1D, S3A,B). Heart weight/body weight (HW/BW) ratio or HW/tibia length (HW/TL) ratio was not increased in RFP+/+ mice at 2 months (Figure S2B,C), but significantly increased in waning mice at 5 months. Heart failure markers such as NPPB was significantly increased in left ventricle (LV) of RFP+/+ mice at 2 and 5 months and in RFP+/− mice at 5 and 12 months (Figure 1D). Because RFP mice remain normotensive (Figure S2D), hypertension was not involved in the hypertrophy of RFP mouse. These results indicate that hypertrophy and heart failure was caused by DsRed tetramers and the severity and the time to onset correlated to the DsRed expression level and ages. In contrast, CALELD+/+ mice, which express high level of EGFP, remained free of any cardiovascular symptom and lived for at least 30 months.

### DsRed tetramer aggregation in cardiac tissues

**Figure 2:**
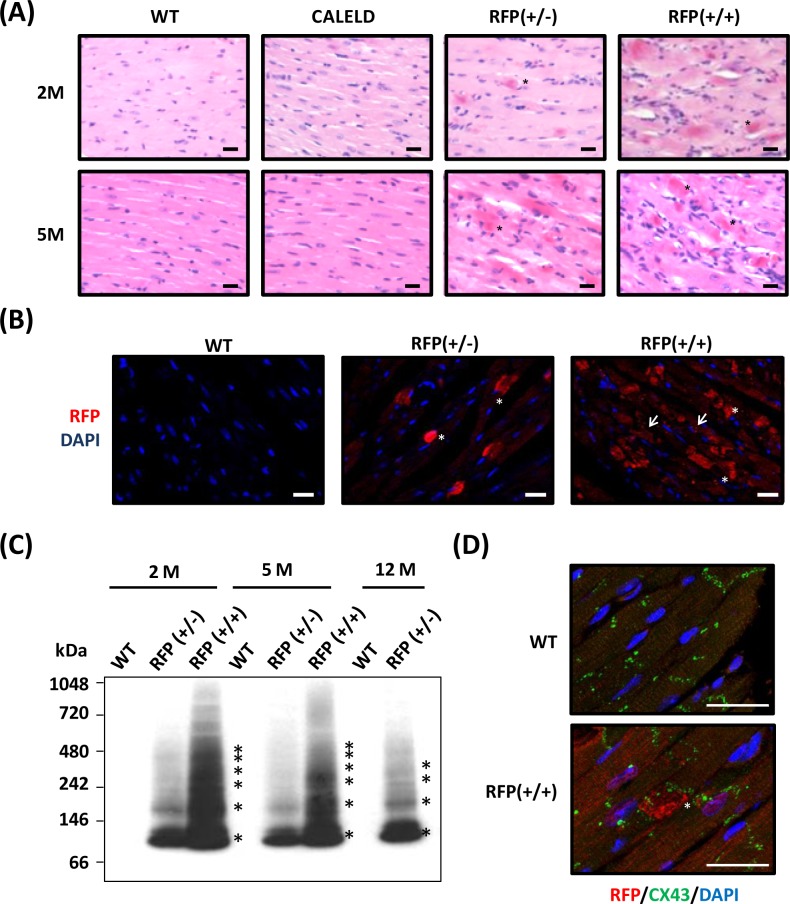
DsRed expression resulted in aggregation and cardiac myopathy **A.** Representative H&E staining of the heart sections isolated from 2-month-old (2M) and 5-month-old (5M) WT, CALELD(+/+), RFP(+/−) and RFP(+/+) mouse. Scale bar = 20 μm. **B.** RFP immunostaining (shown in red by using Alexa Fluor 568-conjugated secondary Ab) and DAPI nuclear staining (blue) in 2-month-old wild-type and RFP heart sections showing large RFP clusters (asterisks) in RFP+/− heart. Both RFP clusters (asterisks) and additional smaller aggregates (arrows) were present in RFP+/+ heart. Scale bar = 20 μm. **C.** Native PAGE and immunoblot using anti-RFP Ab shows the banding pattern of DsRed oligomers in LV of RFP mice. Molecular weights (in kDa) are indicated on the left, and asterisks indicate major DsRed oligomers. MW of DsRed monomer is approximately 28 kDa. **D.** Immunostaining of RFP (red), Connexin 43, (green) and DAPI (blue) of 2- month-old wild-type and RFP+/+ heart. Scale bar =25 μm.

In RFP mouse heart sections the presence of diffused red dots, which were strongly stained by eosin in the cytoplasm of cardiac myocytes was noticed (Figure 2A). Inflammation was found at the surrounding of the cardiac myocytes containing the red dots. The strong eosin-stained cytoplasm was highly reactive to anti-DsRed antibody (Figure S4A). In both 2-month-old RFP+/+ and RFP+/− mouse hearts, immunofluorescence with anti-DsRed antibody revealed large RFP clusters which corresponded to red dots in H&E. Many cardiac myocytes of RFP+/+ heart also contained diffused and smaller RFP patches in addition to large clusters, and these small RFP patches were rarely observed in RFP+/− heart (Figure 2B). This indicates when expressed in low to medium amount as in RFP+/− heart, RFP tends to form large clusters. When expressed in high amount as in RFP+/+ heart, small RFP patches appeared in cardiac myocytes in addition to large clusters. To study RFP forming aggregates or oligomers in mouse heart, LV tissue lysates were analyzed by native electrophoresis followed by immunoblotting to reveal the aggregation and the protein identity. The ladder-like protein bands were reactive to DsRed antibody, and the lowest molecular weight (MW) and each step of protein ladder was about 110 kDa, the predicted MW of a DsRed tetramer (Figure 2C). RFP+/− heart contained less RFP oligomers than the RFP+/+ heart, and the amount of aggregates did not increase with aging. Even in failing RFP+/− heart at 12 months, the RFP+/− aggregates remain not further increased, suggesting that the heart failure may result equally from failed compensation or remodeling and from excessive RFP oligomers. Many large RFP clusters were found at the vicinity of Z-lines, which was stained by connexin 43 (Figure 2D), suggests that DsRed tetramers and high order oligomers may impair cardiomyocyte contractility.

### Impaired protein degradation systems in RFP mouse heart

**Figure 3:**
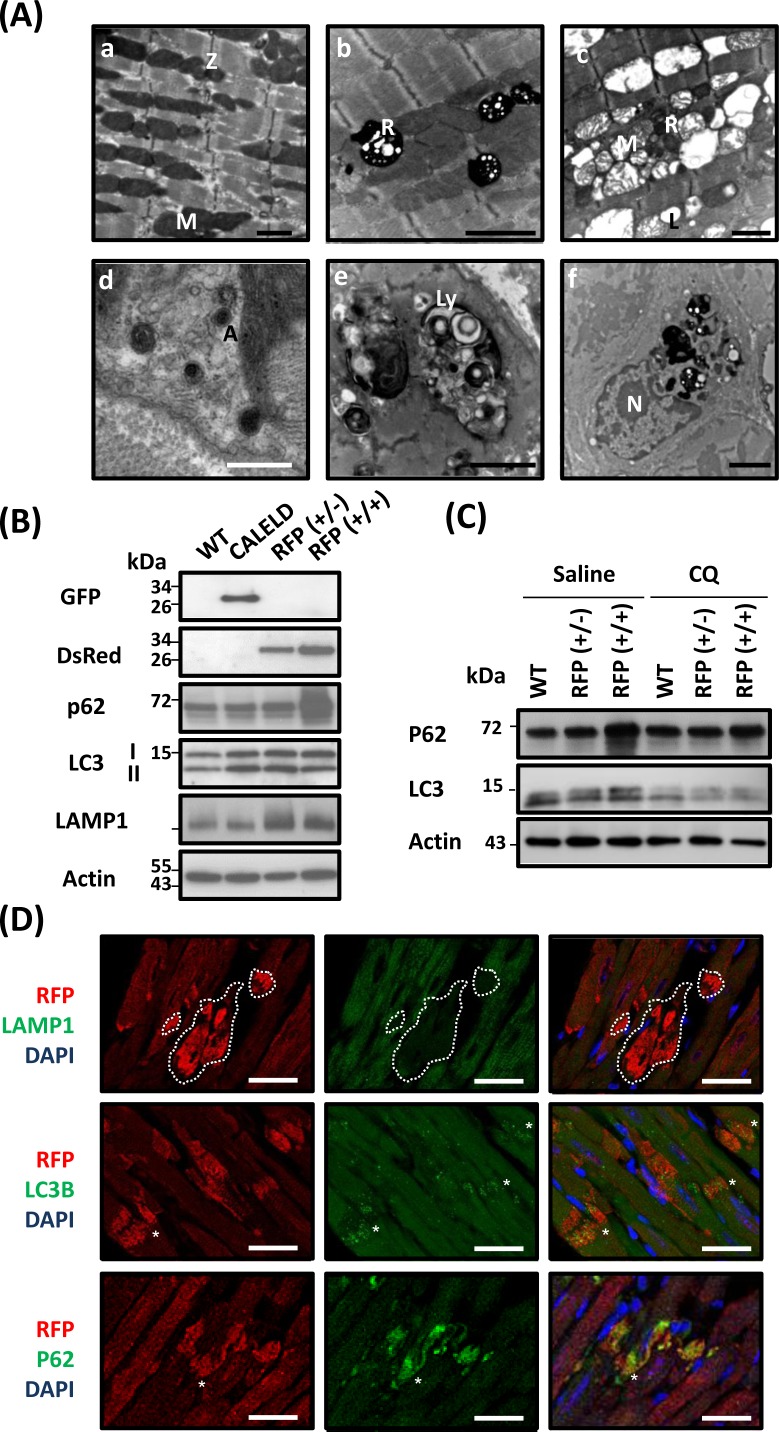
Microstructure defects in RFP mouse heart **A.** Microstructural features of LV tissue sampled from 2-month-old wild-type and RFP+/+ mouse. (a) Wild-type myocardium shows organized myofilaments with clusters of normal mitochondrion aligned between myofibrils. (b-f) RFP+/+ myocardium: (b) Electron-dense residual bodies were detected among clustered mitochondrion with the contrast reduced. The myofilaments, although remain as organized array, appear less compact than wild-type myofilaments. (c) Widespread vacuoles between interrupted myofilaments. Large vacuoles contain damaged mitochondria. Degradation of mitochondrial matrix, disruption of cristae and lipid droplets was also shown. (d) Autophagic vacuoles with typical double membranes contains cytoplasmic material. (e) Large vacuoles of autophagosome (or lysosome) engulfed material of heterogeneous origins. (f) Macrophage in which the cytoplasm contains phagosomes, residual bodies and lysosomes with digested materials. A, autophagosome; L, lipid droplet; Ly, lysosome; M, mitochondria; N, nucleus; R, residual body; Z, Z-line. Black scale bar = 2 μm; white scale bar = 0.5 μm. **B.** The protein of EGFP, RFP, p62, LC3-I and LC3-II, LAMP1, and actin in 2- month-old wild-type, CALELD, RFP+/−, and RFP+/+ heart. Representative images are shown. **C.** p62 and LC3 in wild-type, RFP+/−, and RFP+/+ heart at 4 h after receiving intraperitoneal injection of 10 mg/Kg chloroquine (CQ) or saline. Representative images are shown. **D.** RFP/LAMP1/DAPI, RFP/LC3B/DAPI, and RFP/p62/DAPI triple staining on 2-month-old RFP+/+ heart sections. Top, white dashed line circled a large area containing large RFP aggregates but absent of LAMP1 staining. In contrast, LC3B and p62 are mostly colocalized with large DsRed aggregates (asterisks). Scale bar = 25 μm.

**Figure 4:**
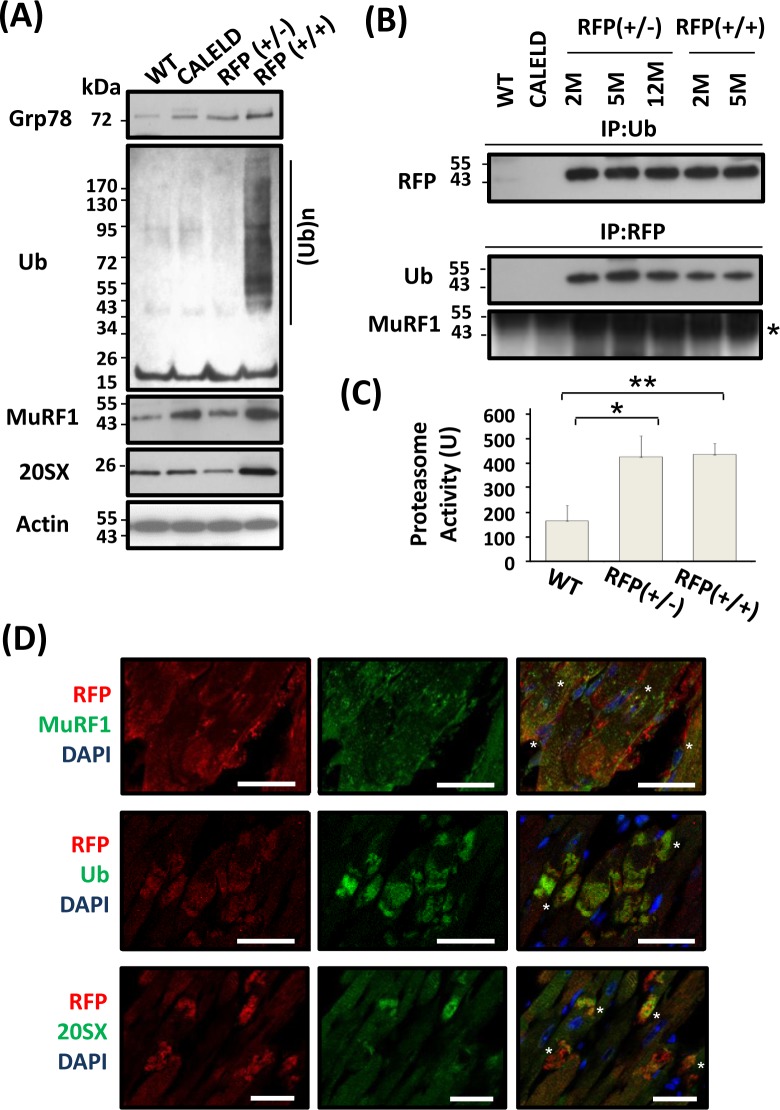
UPS involved in RFP degradation **A.** Grp78, MuRF1, 20SX, actin, and ubiquitinated proteins in 2-month-old wild-type, CALELD, RFP+/−, and RFP+/+ heart. **B.** Immunoprecipitation results showing ubiquitination of DsRed protein in RFP heart lysates by first precipitating with anti-ubiquitin Ab (IP:Ub) and detecting by anti-RFP Ab, or by first precipitating with anti-RFP Ab (IP:RFP) and detecting by anti-Ub Ab or anti-MuRF1 Ab. MuRF1 band (asterisk) is shown. The dark protein bands which were across all samples and located above MuRF1 are considered as non-specific bands. **C.** Increased proteasome activity in RFP+/− and RFP+/+ heart. The proteasome activity was determined as described in the method. *N* = 3. **D.** RFP/MuRF1/DAPI, RFP/Ub/DAPI, and RFP/20SX/DAPI triple staining on RFP+/+ heart sections. Top, MuRF1 and RFP were colocalized at a few areas (asterisks), while remain not associated at other places. Middle, Ubiquitin and RFP were colocalized together, especially at large RFP clusters (asterisks). Bottom, 20SX stained vehicles located at perinuclear locations, and some vesicles colocalized with large RFP clusters (asterisks). Scale bar = 25 μm.

Microstructure of LV of 5-month-old RFP+/+ mouse revealed disarrayed myofibrils with damaged and disintegrated mitochondrion in addition to many residual bodies and vacuoles found between muscle filaments (Figure 3A). These residual bodies are likely part of lysosomal organelles and autophagic vacuoles that are enclosed by the typical double membranes and contained engulfed mitochondrion and cytoplasmic material. We also observed inflammatory cells engulfing residual bodies, vacuoles, and debris (Figure 3A). Because the electron microscopic result showed excessive autophagic vacuoles and lysosomes in failing cardiac tissue, we studied the expression profile of genes that were significantly altered from age-matched wild-type mouse and are relating to autophagy, unfolded protein response (UPR) and ubiquitin pathways (Figure S4B,C). We assumed that a mild increase or decrease in gene expression in 2-month-old RFP+/+ mice followed by a greater change in 5-month-old RFP+/+ mice would indicate the altered gene expression correlating to the severity of phenotypes, thus more likely to be the primary gene response to RFP expression. The genes relating to ubiquitin pathways, including ubiquitin-activating enzymes ubiquitin ligases of E3 (), were increased in the RFP mouse heart. E3) and Lys-63-specific deubiquitinase () were decreased (Figure S4B). The genes including p62/SQSTM1, ATG4C, RGS19 and DRAM1 were increased, and ATG9A was decreased in the RFP mouse heart (Figure S4C). The genes relating to UPRDnaJ which is related to protein folding and endoplasmic reticulum-related genes including ERO1L, ERO1LB, SERP1, DNAJC10, EDEM1 were increased in RFP mice at late stage, but HSPA1l and RNF5, which also plays a key role in protein ubiquitination was reduced (Figure S4B). The protein level of Grp78, markers of ER stress, was increased in RFP mouse (Figure 4A), indicating the elevated ER stress may be involved.

To clarify mechanisms behind early pathological changes leading to myocyte injury, we focused on 2-month-old mice before phenotypes of the later stage occurred. An increase of LC3-I and LC3-II was observed in CALELD, RFP+/−, or RFP+/+ hearts, and LAMP1 was increased in RFP hearts. Further, p62 was strongly induced only in RFP+/+ heart but not in others (Figure 3B). These results suggest that autophagy flux may be altered in RFP+/+ heart. The increased autophagosome-like organelles and increased LC3-II would indicate the likelihood of enhanced autophagy flux, while the accumulation of ubiquitinated proteins and p62/LC3 induction (Figure 4A) were consistent to phenotypes caused by an inhibition of autophagy pathway. Thus, we studied the autophagy flux in RFP heart by injecting mice with chloroquine that would block lysosomal degradation [16] and with monodansylcadaverine (MDC), a fluorescent dye for endosome, autophagosome and lysosome [16, 17]. Chloroquine injection to RFP mouse led to a reduction of p62 and LC3-I and -II (Figure 3C). This result was validated by immunofluorescence staining showing decreased p62 and LC3 labeling in chloroquine-injected RFP mouse heart (Figure S5). In contrast, the intensity of MDC staining and the MDC-stained vacuoles were slightly increased by chloroquine (Figure S5). The result of p62 and LC3 response to chloroquine suggests that RFP+/+ heart was likely under chronic ALS inhibition. Double staining revealed that LC3B- and p62-labelled vacuoles or dots often colocalize with large RFP clusters (Figure 3D), indicating that RFP degradation involves both LC3 and p62. To study RFP in lysosomes, we used the isolated skeletal myoblasts from RFP mouse to investigate intracellular distribution of RFP with the lysotracker, and discovered RFP unequivocally colocalized with lysotracker (Figure S4D). Although in heart sections we failed to find large RFP clusters as part of lysosomal structures (Figure 3D), at least RFP can be located in the lysosome compartments in cultured myoblasts thus likely is degraded by ALS.

Hallmarks of proteinopathy in the cell include an accumulation of ubiquitinated proteins. Levels of ubiquitinated proteins in RFP+/− or CALELD mouse heart were not different from wild-type mouse, while the accumulation of ubiquitinated proteins was found in RFP+/+ heart (Figure 4A). Immunostaining revealed the majority of ubiquitinated proteins colocalized with large RFP clusters (Figure 4D). The expression of MuRF1 increased in CALELD and RFP+/+ mouse heart, and the MuRF1 staining showed some but not all MuRF1 colocalized with RFP (Figure 4A). One subunit of 20S proteasome, 20SX(β5), significantly increased only in RFP+/+ heart, and the staining showed the proteasomes at the predicated perinuclear locations, and were also colocalized with large RFP clusters (Figure 4D). These findings indicate that RFP may be ubiquitinated in cardiac tissue and cleared by UPS. We found in the heart lysate that RFP is ubiquitinated and physically associates with MuRF1 by immunoprecipitation and immunoblotting (Figure 4B). Although RFP expression in RFP+/+ heart lysate was significantly greater than in RFP+/− heart, the amount of ubiquitinated RFP in RFP+/− and RFP+/+ hearts were interestingly similar, indicating possibly a fixed ubiquitination capacity in cardiac tissues available for degrading RFP. Further, we measured the proteasome activity in LV and found the proteasome activity significantly increased in RFP hearts (Figure 4C).

### DsRed aggregates result in cardiac interstitial fibrosis

**Figure 5:**
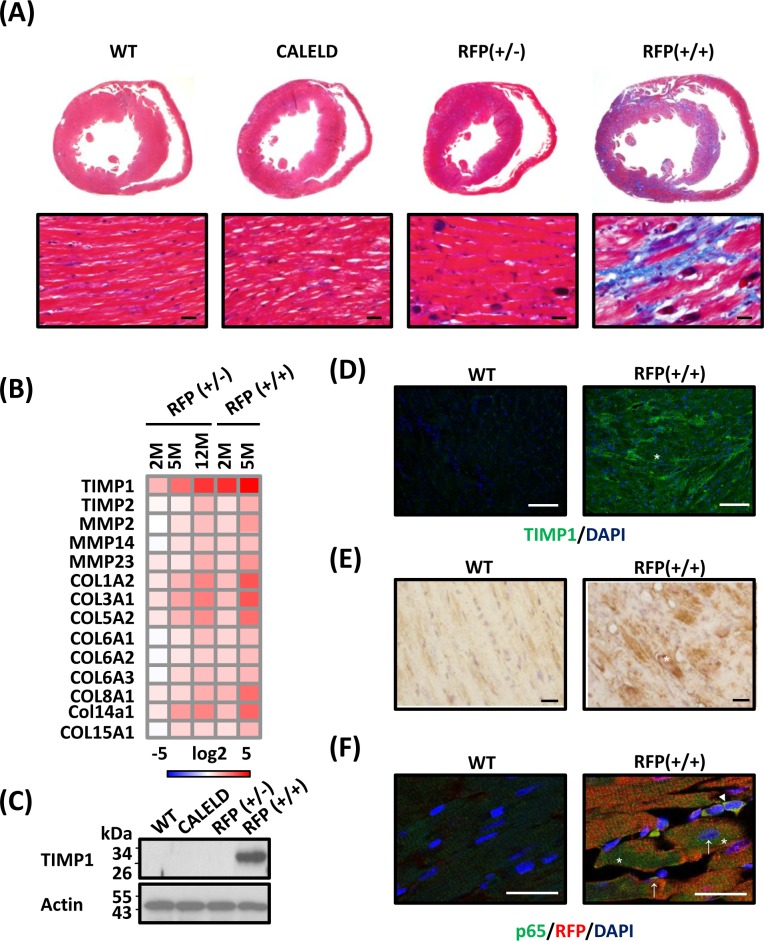
Cardiac fibrosis in RFP mouse **A.** Transverse sections of 5-month-old WT, CALELD+/+, RFP+/− and RFP+/+ mouse heart stained by Masson's trichrome. Red, cardiac tissue; black, nuclei; blue, fibrotic tissue. Scale bar = 20 μm. **B.** ECM-related genes differentially expressed in RFP heart. RNA isolated from the LV of RFP+/−, RFP+/+, and wild-type mouse was analyzed by DNA chip. Each gene is normalized to the wild-type mouse of the same age and the fold changes in log2 are shown. Only differentially expressed genes with P<0.05 (ANOVA) are shown. *N* = 3. **C.** TIMP1 proteins in LV of 2-month-old WT, CALELD+/+, RFP+/−, and RFP+/+ mouse. **D.** TIMP1 expression in 2-month-old wild-type and RFP+/+ heart sections. TIMP1 (green) and DAPI (blue) is shown. TIMP1 expression in cardiac myocytes is also shown in Supplemental Data. Scale bar = 50 μm. **E.** Staining of reactive oxygen species (ROS) in heart sections from 5-month-old WT and RFP +/+ mouse. Scale bar = 20 μm. **F.** p65/RFP/DAPI triple staining on 2-month-old wild-type and RFP+/+ heart. In wild-type heart p65 staining was very weak. In RFP+/+ heart, p65 staining was increased at cytoplasm (asterisks) and at perinuclear locations (arrows). Some cells contained strong p65 staining at nucleus (arrow head) may not belong to cardiac myocytes. Scale bar = 25 μm.

Continuous expression of DsRed tetramers in RFP mice led to extensive fibrosis in late-stage hypertrophied RFP hearts, in contrast to an absence of hypertrophy or fibrosis in CALELD mouse (Figure 5A). In other experimental models most fibrotic lesions occurred at endocardium, epicardium, and perivascular regions [18–20]. In addition to those lesion-prone areas, fibrotic deposits in RFP mouse occurred predominantly at the myocardium. Genes relating to tissue remodeling and fibrosis were highly induced in RFP mice, including TIMP1, MMP2, MMP9, COL1A2, COL3A1, COL8A1 and COL14A1 (Figure 5B). COL1A1, an important marker for cardiac hypertrophy and pathological remodeling, increased significantly in RFP heart (Figure S6A). Besides TIMP1 gene induction, TIMP1 proteins increased significantly in young RFP heart (Figure 5C) and were located within cardiac myocytes and extracellularly (Figure 5D, S6B). The staining for reactive oxygen species (ROS) strongly increased in LV of RFP+/+ mouse at 5 months (Figure 5E), but similar ROS induction was not detected in 2-month-old RFP+/+ or RFP+/− heart (data not shown), suggesting that ROS which is primarily caused by mitochondrial dysfunction may not play the most important role in early pathological changes, but is likely involved in remodeling at the late stage. Both ECM accumulation and ROS induction contributed to continuing remodeling, hypertrophy and extensive interstitial fibrosis. We investigated the potential mechanism by which activates TIMP1 induction in RFP cardiac myocytes. TIMP1 was shown as a downstream target of NF-κB signaling in which TIMP1 induced by the activation of NF-κB signaling in cancer models [21, 22] or under UPR and ER stress [23]. Therefore, we performed the staining for NF-κB p65 subunit as an indicator of NF-κB activation in RFP+/+ cardiac myocytes and found an increase of p65 nuclear and perinuclear staining in cardiac myocytes (Figure 5F). Although a direct causal relation of NF-κB activation in RFP heart and TIMP1 induction was not shown by us, TIMP1 induction together with mitochondrial dysfunction, activation of collagen genes, and ROS production work in sync toward depositing ECM and precipitating hypertrophy.

### Resistance to DsRed-induced proteinopathy in skeletal muscles

**Figure 6:**
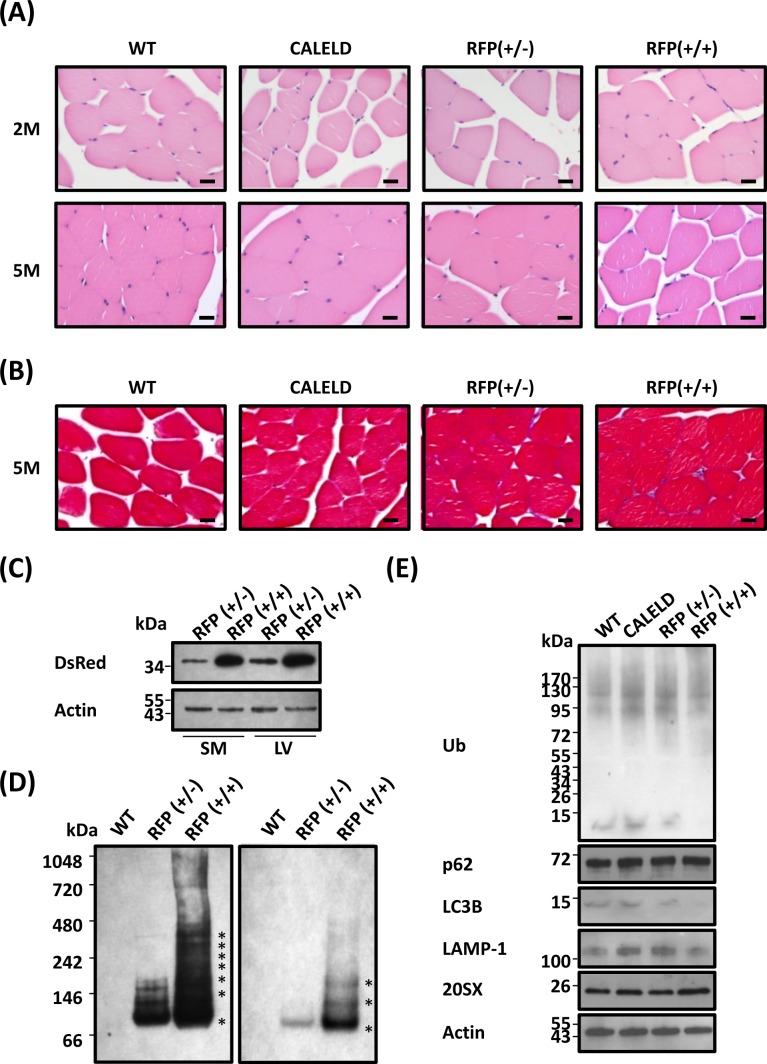
RFP aggregation in skeletal muscles without causing myopathy **A.** H&E staining of skeletal muscle transverse sections isolated from 2-month-old (2M) and 5-month-old (5M) WT, CALELD +/+, RFP+/−, and RFP+/+ mice. Scale bar = 20 μm. **B.** Masson's trichrome staining of skeletal muscle sections of 5-month-old WT, CALELD (+/+), RFP (+/−) and RFP (+/+) mice. Scale bar = 20 μm. **C.** The protein of RFP in the skeletal muscle (SM) and LV of 2-month-old RFP+/− and RFP+/+ mice showing a similar level of RFP expressed in both organs. Representative SDS-PAGE and immunoblotting results are shown. **D.** Native PAGE and immunoblotting as in Figure 2C for detecting DsRed oligomers in skeletal muscle lysates. The gel picture on the right is a lighter exposure of the left picture for showing locations of major bands. **E.** The protein of EGFP, ubiquitinated proteins, p62, LC3B, LAMP1, and 20SX expression in the skeletal muscle of 2-month-old WT, CALELD+/+, RFP+/− and RFP+/+ mice.

Because the CAGGS promoter expresses a high level of transgene in skeletal muscles, we turned to look into any skeletal phenotype in RFP mouse. Skeletal muscle of RFP mouse did not display signs of inflammation, darkened cytoplasm or interstitial fibrosis (Figure 6A, 6B). We thus speculated the amount of transgene expressed in the skeletal muscle or the degree of DsRed oligomerization to be different from that in cardiac muscle. However, the level of RFP expression in skeletal muscles and the amount of RFP oligomers were found very similar to cardiac muscle (Figure 6C, 6D). Accumulation of ubiquitinated proteins was absent in skeletal muscle, indicating a lack of proteinopathy (Figure 6E). In RFP mouse the level of p62, LC3B or LAMP-1 in skeletal tissues was not different from the wild-type mouse.

### Heart-specific tetrameric DsRed expression led to heart failure

**Figure 7:**
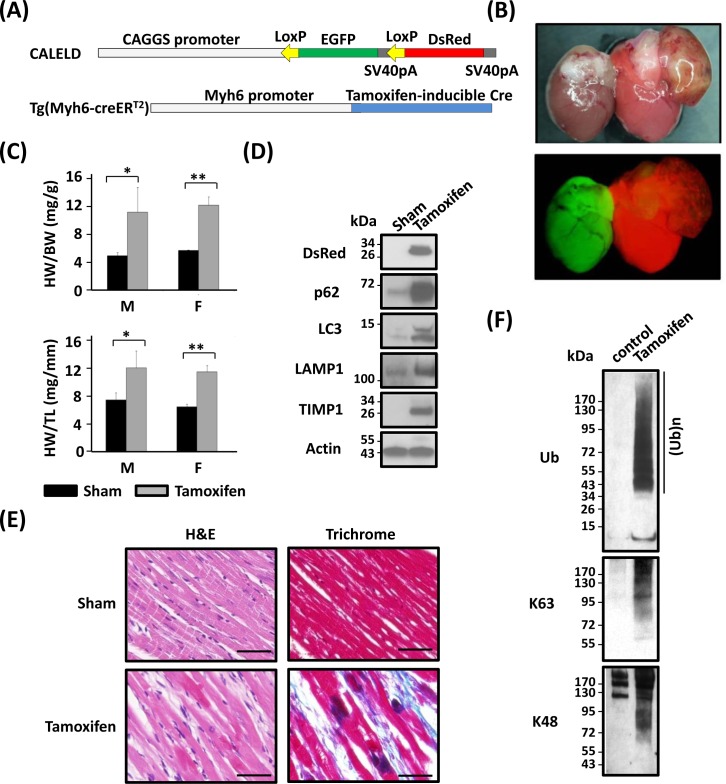
Heart failure resulted from cardiac-specific RFP expression **A.** CALELD and Myh6-creERT2 double-Tg mice. **B.** Bright-field (top) and fluorescent (bottom) images of hearts from double-Tg mice as sham control (left) or tamoxifen-induced (right) showing the color change from green to red by feeding with tamoxifen-contained chow. **C.** HW/BW and HW/TL of double-Tg mice which were sacrificed at 10 weeks after receiving tamoxifen or control chow (sham). **, *P* < 0.01; *, *P* < 0.05 compared with the sham group. The number of mice used in this study: Sham (M 4, F 3); Tamoxifen (M 4, F3). **D.** T RFP, p62, LC3B, LAMP1 and TIMP1 in the heart of double-Tg mice receiving tamoxifen or of sham control. **E.** H&E and trichrome staining of the hearts of tamoxifen-induced or of sham control. Scale bars = 50 μm. **F.** Ubiquitinated proteins in the hearts of tamoxifen-induced or of sham control. Ub, pan ubiquitination; Ab specific for K63-, or K48- ubiquitination.

We generated Myh6-CreERT2:;CALELD+/+ double-Tg mouse to achieve expressing DsRed transgene only in cardiac tissues (Figure 7A) to confirm our findings and exclude causes such as RFP accumulation in other organs that could have contributed to heart dysfunctions. Following the tamoxifen treatment (feeding mice with tamoxifen-containing chow for 2 weeks), severe respiratory stress was developed in double-Tg mice which had to be euthanized by 10 weeks after initiating tamoxifen diet (Figure 7B). Cardiac phenotypes including hypertrophy, left atrium thrombosis, and interstitial fibrosis were similar to those observed in the ubiquitous RFP mice. An increase of HW/BW and HW/TL ratios was resulted in tamoxifen-induced mice (Figure 7C). Because control Tg mice such as Myh6-CreERT2 mice or CALELD mice fed by tamoxifen-chow did not develop hypertrophy (data not shown) and that tamoxifen treatment was for only two weeks, tamoxifen by itself unlikely caused the heart failure. An increase of p62, LC3B, LAMP1 and TIMP1 proteins was found in tamoxifen-induced heart (Figure 7D). Similar dark spots by H&E staining and extensive interstitial collagen deposits were present in tamoxifen-induced double Tg mouse heart (Figure 7E). We observed a massive accumulation of ubiquitinated proteins including proteins with the ubiquitin attached at residue Lys-63 or Lys-48 (Figure 7F), indicating the ubiquitin-proteasome stress in affected heart. Taken together, the CV phenotypes induced by cardiac-specific RFP expression agree with RFP mouse and that DsRed tetramer aggregation in heart is sufficient to induce proteinopathy and congestive heart failure.

## DISCUSSION

In this study, we generated the RFP mouse by recombining the transgene of CALELD mouse, which serves as an ideal control for the RFP mouse because the promoter, transgene copies, level of expression, and the location of genome insertion were the same for both mice. EGFP, which primarily forms monomers or dimers, is less toxic to the cells than DsRed. That a massive GFP expression led to hypertrophic and dilated heart failure in a transgene copy-dependent manner was previously reported [24], but we did not observe any CV phenotype in CALELD mice. One possible explanation is that the amount of EGFP expressed in CALELD mice is less than the previous model. EGFP that binds to the myosin and interrupts the cytoskeletal component has the potential to disrupt the contractile function of muscle cells [25]. The toxicity of EGFP aggregation was demonstrated by various cell culture models [26–28], including that EGFP-induced toxicity was related to an inhibition of NFμB signaling and to a suppressed protein ubiquitination and degradation [27, 29, 30]. By using CALELD and RFP Tg mouse, we show protein ubiquitination not affected by EGFP and that RFP expression led to an increase, instead of a decrease, of protein ubiquitination in cardiac myocytes.

Tetrameric DsRed, one of early RFPs developed for molecular imaging applications, was replaced by newer monomeric RFPs. Tetrameric DsRed was reported to suppress Bcl-xL translation [14]. Previously, the toxicity of tetrameric DsRed to embryonic and hematopoietic stem cells prevented the generation of Tg mouse expressing DsRed tetramers [15, 31, 32]. The DsRed monomer Tg mice were later generated without any reported abnormalities [32–34]. While the toxicity of DsRed tetramers in cell culture was known for a long time, without DsRed mice, the *in vivo* consequence by DsRed-elicited toxicity in whole body and a detailed pathological analysis was not previously feasible. In this work, we demonstrate for the first time in animal model the proteinopathy caused by aggregation of DsRed tetramers.

With the advance of technology for diagnoses and increasing awareness, heart diseases caused by proteinopathy are likely to increase in the future. Proteinopathy is the primary cause of senile cardiac amyloidosis and of certain congenital heart diseases such as desmin-related cardiomyopathy (DRC) [35–37]. Phenotypes of RFP mouse including aggregation, inflammation, massive ubiquitinated proteins and DCM were consistent with phenotypes caused by chronic inhibition of autophagy or by defective proteasomal function [38, 39]. By analyzing the cardiac transcriptome at various ages and of various levels of expression, we found in RFP heart that genes with the most changes which correlate impressively with the severity of myopathy are groups of genes relating to cardiotoxicity (Table S1). RFP mouse displays histology features similar to that of amyloidosis or DRC. Heart sections of amyloidosis sometimes show typical apple-green birefringence under polarized light with Congo red staining. Fluorescent proteins contain abundant β-sheet structures, but we did not observe similar birefringence in RFP heart sections (data not shown) probably due to the nature of DsRed aggregates not stainable by Congo red.

Induction of p62/SQSTM1 and massive ubiquitinated proteins are the most noticed features in young RFP mouse heart. p62 is involved in both proteasome-mediated proteolysis and autophagosome/lysosome-mediated protein degradation [40]. Accumulation of p62 was commonly found in cardiac UPR or proteasome functional insufficiency (PFI) models that suggests a pivoting role by p62 in mediating UPR and PFI-induced myopathy [41]. The origins of p62 induction in proteinopathy may include an enhanced de novo p62 synthesis, an inhibition of autophagy that is responsible for p62 degradation, or by selective autophagy of damaged organelles and protein aggregates [42]. p62 induction in RFP heart can be addressed from two perspectives : (1) whereas p62 transcripts were moderately increased in both RFP+/+ and RFP+/− heart, p62 protein was not increased in RFP+/− heart. NRF2 signaling, which mediates p62 induction in other models was not induced in RFP heart. Thus, the increase of p62 protein in RFP+/+ heart was probably not due to an increase of de novo p62 synthesis as in other models [43]; (2) we expressed DsRed tetramers in H9C2 cells, a cell line of cardiac origin, and treated cells with chloroquine, which resulted a mild increase of p62 without enhanced protein ubiquitination. Treating cells with bortezomib, a proteasome inhibitor, resulted a strong p62 induction and accumulation of ubiquitinated proteins (data not shown). Thus, p62 induction in RFP heart likely resulted from proteasome stress such as under PFI or due to a reduction of autophagy. In our view, pathogenesis of RFP-induced proteinopathy likely involves both PFI and chronic autophagy/lysosome inhibition and the former likely plays a more important role. We showed the clustered RFP physically associated with ubiquitin, p62, LC3B, and with the MuRF1, which potentially utilized ubiquitin-tagged RFP as a substrate. Clustered RFP were frequently located close to the junctions that likely cause the interruption of contractility. MuRF1 mediates protein degradation at the vicinity of Z-disk, and increased expression of E3 ligases including MuRF1 and MAFbx was considered as markers of muscle atrophy [44, 45]. UPS activation and MuRF1 induction in RFP+/+ heart potentially could trigger an atrophic response, leading to a loss of myocytes, which were subsequently replaced by fibrotic tissues.

Containing similar levels of DsRed aggregation as in cardiac tissues, skeletal muscles tolerate DsRed-induced proteinopathy, and some RFP+/− mice were free of skeletal symptoms throughout the life span of at least 18 months. One explanation for cardiac cells particularly vulnerable to proteinopathy is in that higher oxidative stress experienced by cardiac cells may be a crucial cofactor in producing damages in proteasomes [46]. Other explanations also exist, such as skeletal muscle can tolerate the protein toxicity and myocyte loss due to a better renewal capability than cardiac myocytes.

Most experimental models of cardiac interstitial fibrosis usually develop fibrotic tissues preferentially at the epicardium, endocardium or perivascular regions [47–51]. In contrast, fibrotic deposits were extensively distributed in RFP mouse with the most severe fibrosis occurred in myocardium, suggesting that interstitial fibrosis caused by RFP may result from a distinct mechanism which potentially involves the mechanical loading as a precipitating factor.

Hypertrophy and DCM as myocardial maladaptation to the pathological stress can be induced by a tilted balance between MMPs and TIMPs [52–54]. Elevated MMP2, TIMP1, collagen type I and III levels were reported in patients with hypertrophy and heart failure [55–57]. Collagen expression and TIMP1 induction in RFP heart correlated strongly with transgene dosage and severity of the disease. The changes in MMP levels except for MMP2 or MMP9 were relatively mild, strongly agreeable with those clinical reports and the net balance between TIMPs and MMPs favored ECM deposition. TIMP1 induction started in young RFP mouse heart and continuing activation suggests the potential of TIMP1 as a biomarker for detecting early stage cardiac fibrosis. We identified NF-κB activation and an increase of markers for UPR and ER stress in RFP heart, consistent with previous reports showing NF-κB activation in cardiac myopathy involving ER stress and UPR [58, 59]. TIMP1 induction in RFP heart may involve NF-κB signaling, but to clarify TIMP1 induced directly by NF-κB requires further study. RFP-induced cardiac dysfunctions suggest an important role of the autophagy lysosomal pathway mediating hypertrophy and fibrosis. This is in line with reports showing therapeutic potential to control tissue fibrosis and other aging-related diseases by modulating autophagy pathways and with reports on established roles of mTOR (mammalian target of rapamycin) in regulating protein synthesis, cell proliferation, immune cell activation, and autophagy [60–62]. Inhibition of mTOR with rapamycin or by inhibitors at its upstream or downstream signaling pathways were shown to reduce interstitial fibrosis [60, 63]. Thus, our future work will try to amend the cardiac myopathy in RFP mice by rapamycin or rapalogs.

**Figure 8:**
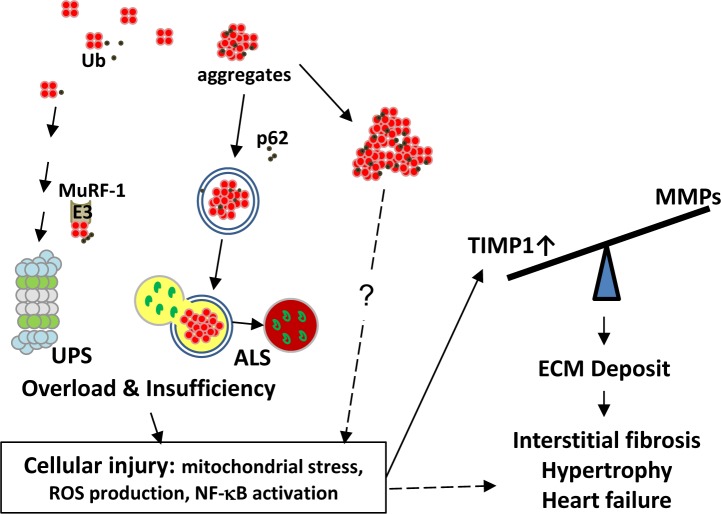
Working hypothesis for the mechanism of interstitial fibrosis and heart failure caused by RFP aggregates **A.** Cellular proteins including RFP in small amount are normally degraded and recycled by UPS and ALS. We identified RFP can be ubiquitinated and MuRF1 as one of the E3 ligases involved in RFP degradation. Excessive amount of DsRed tetramers can overload UPS and ALS, thus deplete its functional reserve for degrading endogenous substrates, causing accumulation of ubiquitinated proteins as a sign of functional insufficiency and eliciting cellular injuries. Large RFP aggregates which usually bound by p62 and LC3 may form aggresome-like structures and cause additional damage to UPS and ALS. These injuries in cardiac myocytes include mitochondrial stress, ROS production and NF-κB activation that potentially lead to TIMP1 and collagen expression as one of many downstream effectors. The tilted balance between TIMPs and MMPs augments deposition of ECM, leads to fibrosis, hypertrophy and heart failure. However, similar proteinopathy is not observed in the skeletal muscle cells which tolerate the RFP aggregates by mechanisms still unclear to us. ALS, autophagosome-lysosome system; Ub, ubiquitin; UPS, ubiquitin-proteasome system.

In summary, main findings of this study include the following: (1) transgenic expression of tetrameric DsRed caused myocardial injury, fibrosis and heart failure in RFP mouse. S of RFP mouse contained comparable levels of aggregates as in cardiac tissues, but remained symptom free; (2) RFP aggregates increased the loading to the UPS and the ALS and ultimately resulted both systems overloaded with pathology appearance similar to that of PFI and of chronic ALS inhibition. Other pathology features include the accumulation of residual bodies, mitochondrial dysfunction, and ROS production that leads to myocyte injury and death; and (3) cell injury and necrosis triggers inflammation and TIMP1 induction, leading to massive interstitial fibrosis, pathological hypertrophy, and DCM. Our working hypothesis for the heart failure in RFP mouse is summarized in Figure 8. Taken together, we demonstrate here the RFP mouse as a model of proteinopathy-induced DCM. This model can be used as a platform for *in vivo* testing of new pharmaceuticals and/or for developing the novel treatment of senile cardiac diseases.

## MATERIALS AND METHODS

### Transgenic mice

Generation of FVB/NJ CAGGS-LoxP-EGFP-LoxP-DsRed (CALELD) and Cre transgenic mice has been described previously [64, 65]. Myh6-creER^T2^ transgenic mice were obtained from the Jackson laboratory (Bar Harbor, ME, USA). CALELD mice were crossed to Cre mice to generate DsRed-expressing mice. Heterozygous or homozygous RFP mice without the Cre allele were subsequently generated. CALELD mice and Myh6-creER^T2^ mice were used to obtain a double-Tg mouse. A tamoxifen diet (*LAS TAM400*, *LASvendi, Germany*) for two weeks was used to induce recombination. Tail DNA was purified using an Allele-In-One Mouse Tail Direct PCR System kit (Allele Biotech, San Diego, USA). For the CALELD allele, the forward and reverse primers were 5′-CTGCTAACCATGTTCATGCC-3′ and 5′-GTACTGGAACTGGG GGGACAG-3′. For the DsRed allele, the forward and reverse primers were 5′-CTGCTAACCATGTTCATGCC-3′ and 5′-GATCTGGAACTGGGGGGACAG-3′. All animal experiments were conducted by conforming to the NIH guideline (Guide for the care and use of laboratory animals) and approved by National Health Research Institutes, Taiwan [NHRI-IACUC-099011A, NHRI-IACUC-100124A].

Additional Materials and Methods can be found in the Supplemental Data.

### Statistics

All values are expressed as the mean ± standard error mean (SEM). Data were analyzed using the Student's *t*-test and *P* < 0.05 was considered significant.

## SUPPLEMENTARY MATERIALS FIGURES AND TABLES


