# Supplementary material for: Cardiac fibrosis in mouse expressing DsRed tetramers involves chronic autophagy and proteasome degradation insufficiency
Source: Oncotarget. 2016 Aug 2;7(34):54274–89. doi: 10.18632/oncotarget.11026 (PMC5342341; doi:10.18632/oncotarget.11026)
Supplement: Supplementary file 1 [file oncotarget-07-54274-s001.pdf]

Cardiac fibrosis in mouse expressing DsRed tetramers involves chronic autophagy and proteasome degradation insufficiency

1. Supplemental Materials and Methods

Protein analysis

For SDS-PAGE, LV and skeletal muscles from Tg mice were homogenized in RIPA buffer (Amresco, OH) using a protease inhibitor cocktail (Roche, Basel, Switzerland). The lysates were collected and quantified by BCA protein assay kit (Thermo, IL). The extracted proteins were separated by 4-15% gradient gels (Mini-Protean TGX^{TM}, BioRad). For native protein gel electrophoresis, LV and skeletal muscle from Tg mice were homogenized in detergent-free buffer with protease inhibitors and separated using NativePAGE Novex Bis-Tris Gel System (Invitrogen, MA). After transfer to a nitrocellulose membrane, primary antibodies, corresponding secondary antibodies and ECL plus reagents (Millipore, Darmstadt, Germany) were used in immunoblotting. The following antibodies were used: GFP (Rockland 600-101-215, PA), DsRed (Clontech 632496, CA), LC3B (Cell Signaling 2775**,** MA), LAMP1 (Abcam ab25245, Cambridge, UK), TIMP1 (Thermo MA1-773), MuRF1 (GeneTex GTX24125, CA), 20SX (GeneTex GTX23330, CA), ubiquitins (Cell Signaling 3933; K48, 4289; K63, 5621), p62/SQSTM1 (Sigma P0067, MO), Grp78(Genetex GTX113340, TX), and β-actin (Millipore MAB1501).

mRNA isolation, GeneChip hybridization and reverse transcription-PCR analysis

Total RNA was extracted from the LV of Tg mice at 2 months, 5 months and 12 months of age using the TRIzol reagent (Invitrogen). RNA quantity and purity was determined by gel electrophoresis and spectrophotometry. Microarray service was provided by Phalanx Biotech (Hsinchu, Taiwan) using Mouse OneArray Express^{TM} with three samples per group, and each sample was analyzed in triplicate. The selection criteria for identifying differentially-expressed genes in the Tg mouse samples compared with the same age of WT mouse are established at log2 (fold change) $\geq$ 1.0 and $P < 0.05$. For qPCR analysis, first-strand cDNA was synthesized using an oligo (dT) primer (ReverTra Ace reverse transcriptase, Toyobo) and qPCR was performed by ABI7900 (Applied Biosystems, MA) using SYBR-Green and the gene-specific probes as follows: NPPB (BNP, natriuretic peptide B) gene, forward primer 5′-GTCAGTCGTTTGGGCTGTAA-3′, reverse primer 5′-AAGAGACCCAGGCAGAGTCA-3′ [1]; Col1A (procollagen α1) gene, forward

primer 5′-CAGATTGAGAACATCCGCAGCC-3′, reverse primer 5′-TGA CCTGTCTCCATGTTGCAGTAG-3′; and glyceraldhyde-3-phosphate dehydrogenase (GAPDH) forward primer 5′-GTGGCAAAGTGGAGATTGTTGCC-3′, reverse primer 5′-GATGATGACCCGTTTGGCTCC-3′ [2].

Histological analysis and immunostaining

Mouse hearts were perfused with PBS and fixed with 10% formalin buffer. Hearts embedded in paraffin were cut into serial 5 μm sections and stained using hematoxylin and eosin (H&E) for histology, and Masson's trichrome, or Martius scarlet blue (MSB) for collagen evaluation. For staining reactive oxygen species (ROS), the OxyIHC™ Oxidative Stress Detection Kit (Millipore) was used to measure protein oxidation in tissue sections. For immunostaining, fixed sections were first blocked and incubated with primary antibodies overnight at 4°C. Antibodies used in immunostaining were RFP (Genetex GTX82561), p62/SQSTM1 (Sigma P0067), ubiquitin (Cell Signaling 3933), MuRF1 (GeneTex GTX24125, CA), 20SX (GeneTex GTX23330, CA), NF-κB P65 (Abcam ab7970, Cambridge, UK), Connexin 43 (GeneTex GTX54818, CA), LC3B (Cell Signaling 2775**,** MA), LAMP1 (Abcam ab25245, Cambridge, UK) and TIMP1 (Thermo MA1-773), in conjunction with a fluorophore-conjugated secondary antibody (Invitrogen). DAPI (Invitrogen) was for staining cell nucleus. Images were taken using an Olympus BX51 microscope equipped with DP72 CCD or by the confocal microscope (Leica TCS SP5 II).

Transmission electron microscopy examination

LV free wall near the apex of heart was fixed in 2.5% glutaraldehyde and 2% paraformaldehyde in 0.1 M sodium cacodylate, post-fixed in 1% Osmium tetroxide, neutralized by ammonium chloride, dehydrated through an acetone series, and embedded in araldite. Thin sections were stained with 2% (w/v) uranyl acetate and 30 mM lead citrate and air dried for later inspection by using a Hitachi H7650 (Japan) or FEI Tecnai G2 spirit electron microscope (FEI, OR).

Zygosity identification in transgenic mice

Genotyping and zygosity determination was performed on tail biopsies using PCR, as described previously [3]. qPCR was performed on an ABI7900 using SYBR®-Green and the gene-specific probes. For the DsRed allele, the forward primer was 5′-CTGCTAACCATGTTCATGCC-3′ and the reverse primer was 5′-GATCTGGAACTGGGGGACAG-3′.

Non-invasive blood pressure measurement in mice

Blood pressure of WT, RFP+/- and RFP+/+ mice was measured weekly from 2 months until 5 months of age using a programmable sphygmomanometer (BP-98A; Softron, Japan) in which the systolic, mean and diastolic blood pressure was measured using the tail-cuff method as described previously [4]. Statistical analysis for the blood pressure was performed using a Student's *t*-test.

Primary skeletal myoblasts isolation and expansion

Myoblasts were isolated from the hind leg muscles of 4-week-old RFP+/+ mouse. Muscle tissue was minced and then treated with collagenase I (GIBSO). Expansion and enrichment of myoblasts was achieved by repeated plating and cultivation in Ham F-10 nutrient mixture (Life Technologies) containing 20% fetal bovine serum (FBS), 2.5ng/ml basic fibroblast growth factor.

Immunoprecipitation Followed by Immunoblot

LV of 2-month-old RFP+/+ mouse were homogenized in RIPA buffer containing the protease inhibitor cocktail (Roche, Basel, Switzerland). 2 μg RFP antibody (Genetex GTX82561) or ubiquitin antibody (Genetex GTX19247) was added to 500 μg total protein lysate and incubate for 1 hour at 4° C, followed by adding 20 μl of resuspended volume of Protein G PLUS-Agarose (Santa Cruz sc-2002). The mixture was rotated overnight at 4°C. The beads were gently washed 4 times using RIPA buffer and each time was spinning down for 5 minutes at 1000g. After wash, proteins were boiled in SDS sample buffer (Bio-Rad) and analyzed by SDS-PAGE and immunoblotting. Antibodies used are DsRed (Clontech 632496, CA), ubiquitins (Cell Signaling 3933), and MuRF1 (GeneTex GTX24125, CA).

Proteasome Activity Assay

Proteasome activity assay was measured by using a Proteasome Activity Fluorometric Assay Kit (BioVision K245-100, CA) and by following the manufacturer's protocol. The kinetics of released substrate fluorescence Succ-LLVY-AMC (Ex/Em = 350/440 nm) was measured in a micro-plate reader at 37 ° C for 30 - 60 min. Proteasome inhibitor MG132 was included in the reference reaction wells for subtracting the non-proteasome related fluorescence. The proteasome-specific activity between the time interval, T1 and T2 is determined as ΔRFU = (RFU2 – iRFU2) – (RFU1 – iRFU1), where RFPU1 and RFU2 is the fluorescence reading without the inhibitor at T1 and T2 respectively, and iRFU1 and iRFU2 is the reading with the inhibitor at T1 and T2. ΔRFU is then converted to the proteasome activity in nmol/min/ml = U/ml by adjusting the lysate volume and following an AMC standard curve.

Autophagic Flux in Live Animals by Chloroquine Injection

Autophagy flux in live mice was measured by adopting a published protocol [5]. Chloroquine (10mg/kg, Sigma C6628), monodansylcadaverine (MDC, 1.5mg/kg, Sigma 30432), or saline was administered through i.p. injections. Four hours after chloroquine injection or one hour after MDC injection, animals were sacrificed and the cardiac tissues were harvested immediately for the lysate collection, or fixed in 10% formalin followed by frozen sections for visualizing MDC and paraffin-embedded sections for LC3B, p62 staining.

References

1. Isensee J, Witt H, Pregla R, Hetzer R, Regitz-Zagrosek V and Noppinger PR. Sexually dimorphic gene expression in the heart of mice and men. Journal of Molecular Medicine. 2008; 86(1):61-74.
2. McCroskery S, Thomas M, Platt L, Hennebry A, Nishimura T, McLeay L, Sharma M and Kambadur R. Improved muscle healing through enhanced regeneration and reduced fibrosis in myostatin-null mice. Journal of Cell Science. 2005; 118(Pt 15):3531-3541.
3. Shitara H, Sato A, Hayashi J, Mizushima N, Yonekawa H and Taya C. Simple method of zygosity identification in transgenic mice by real-time quantitative PCR. Transgenic Research. 2004; 13(2):191-194.
4. Tanimoto K, Sugiyama F, Goto Y, Ishida J, Takimoto E, Yagami K, Fukamizu A and Murakami K. Angiotensinogen-deficient mice with hypotension. The Journal of biological chemistry. 1994; 269(50):31334-31337.
5. Iwai-Kanai E, Yuan H, Huang C, Sayen MR, Perry-Garza CN, Kim L and Gottlieb RA. A method to measure cardiac autophagic flux in vivo. Autophagy. 2008; 4(3):322-329.

2. Supplement Figures and Legends

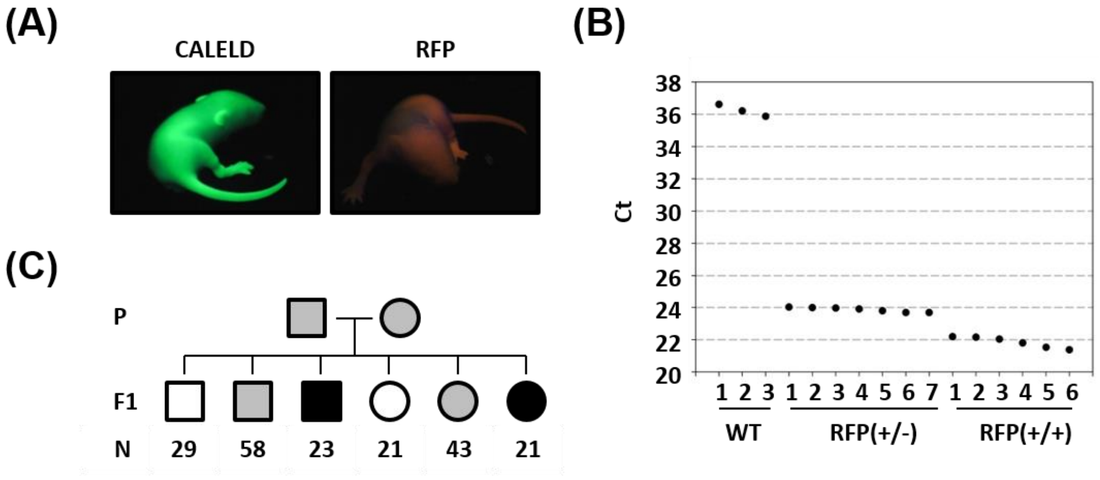

Fig. S1. **Zygosity identification in RFP mice using quantitative PCR**

(A) Pictures of CALELD and RFP Tg mouse.

(B) Threshold cycles (Ct) of quantitative PCR using the tail-DNA from littermates. Ct shown were approximately 22, 24 and 36 for RFP+/+, RFP+/-, and WT mice respectively.

(C) Zygosity of littermates from the crossing of RFP+/- x RFP+/- mice in accordance with the Mendelian frequency. Circles, female; Squares, male; Black, RFP+/+; Gray, RFP+/-; White, Wildtype. P, parents; N, numbers of littermates.

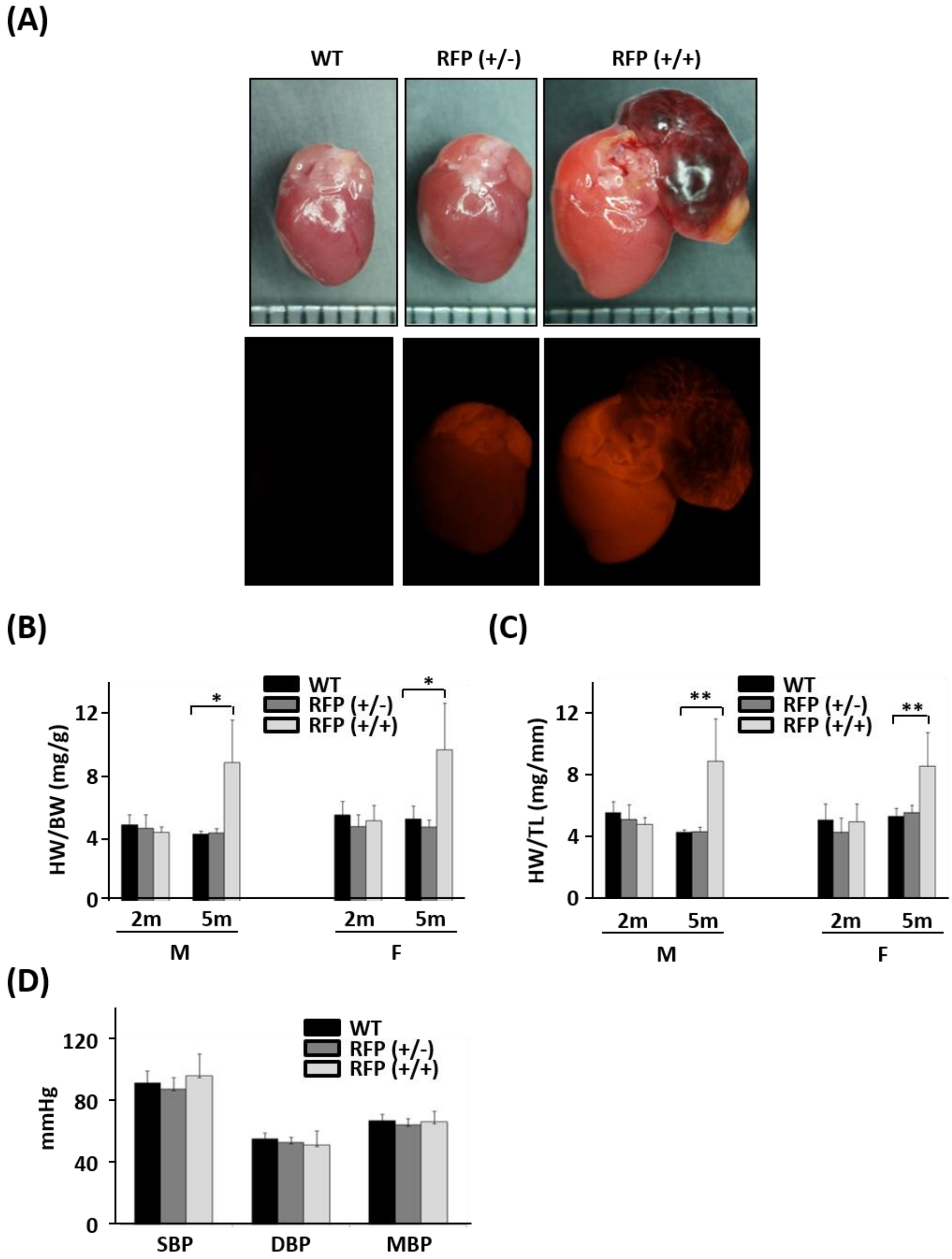

Fig. S2. Ventricular hypertrophy in RFP mice

(A) Bright field (top) and fluorescent images (bottom) of Wildtype WT, RFP+/- and RFP+/+ mouse heart. Scale bar = 1 mm.

(B) (C) Ratios of Heart weight /Body weight (HW/BW) and HW /Tibia Length (HW/TL) of 2- and 5-month-old Tg mice. Data are presented as the mean ± SEM. **, P < 0.01; *, P < 0.05 compared with age-matched wildtype mouse. The number of mice in comparison: 2 month mice, WT (M 8, F 7), RFP +/-

(M 12, F 8), and RFP+/+(M 7, F 7); 5 month mice, WT (M 6, F 6), RFP+/- (M 12, F 9), and RFP+/+(M 4, F 7).

(D) RFP mice remain normotensive at 5 months of age. SBP, systolic blood pressure; DBP, diastolic blood pressures; MBP, mean blood pressure The number of mice used in blood pressure measurement: wildtype mice (M 5, F 4), RFP+/- mice (M 11, F 9) and RFP+/+ mice (M 6, F 11).

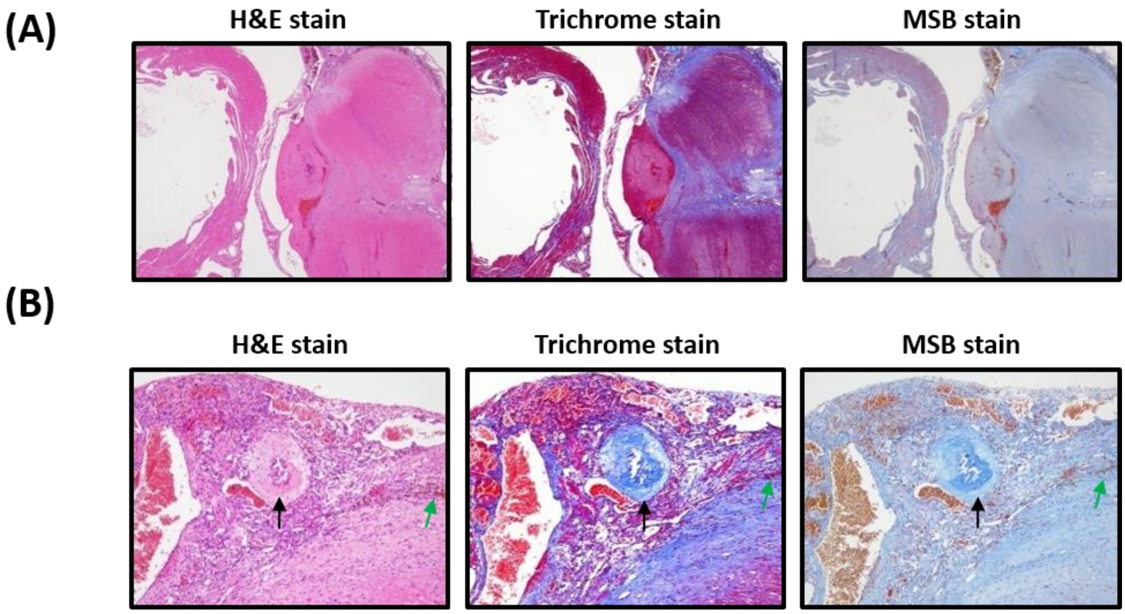

Fig. S3. Left atrium thrombosis in RFP mice

(A) Histochemical analysis of heart sections (representative LA) of 5-month-old RFP+/+ mouse using H&E, Masson's and Martius scarlet blue (MSB) trichrome staining. Masson's trichrome staining: red, cardiac tissue; black, nuclei; blue, fibrosis. MSB staining: light red, cardiac muscle; blue-black, nuclei; blue, collagen and elastic fibrosis; red, fibrin.

(B) Features of dystrophic calcification (black arrows) and hemosiderin (green arrows) in the left atrium of 5-month-old RFP +/+ mouse.

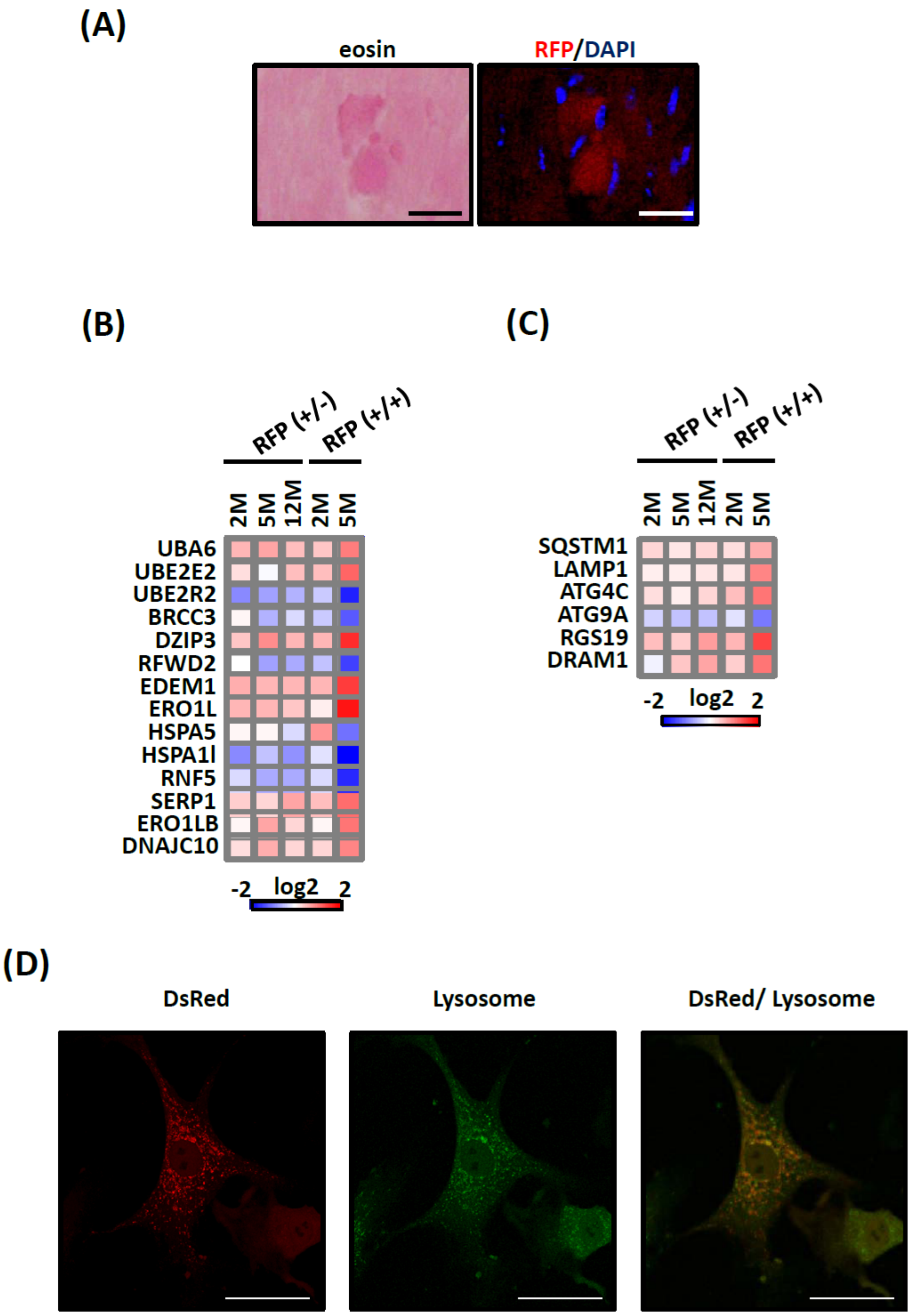

Fig S4.

(A) Eosin and immunofluorescence staining of 2-month-old RFP+/+ heart showing the myocytes with the cytoplasm strongly stained by eosin, appearing as those red dots in H&E pictures (Fig. 2A), also positively stained by Anti-RFP Ab. Alexa Fluor 568-conjugated secondary Ab for RFP and DAPI for nuclear staining was used. Scale bar = 20 µm.

(B) (C) ER stress, UPR, ubiquitination, and autophagy related genes differentially

expressed in RFP mouse heart. RNA isolated from the LV of RFP+/-, RFP+/+, and wild-type mice of various ages was subjected to array analysis. Each gene is normalized to the WT mouse of the same age and the fold changes in log2 are shown. Only differentially expressed genes with $P<0.05$ (ANOVA) are shown. N=3.

(D) Primary RFP+/+ mouse skeletal myoblasts showing colocalized RFP autofluorescence with LysoTracker Green (Invitrogen DND-26), indicating that DsRed is degraded by ALS in myoblasts. Scale bar = 20 μm.

(A)

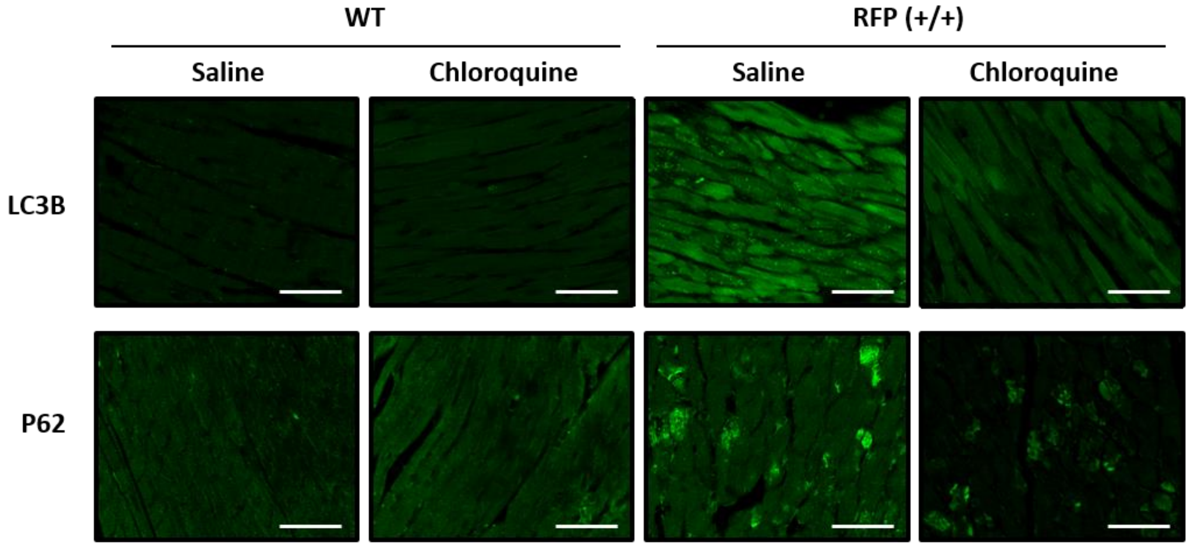

(B)

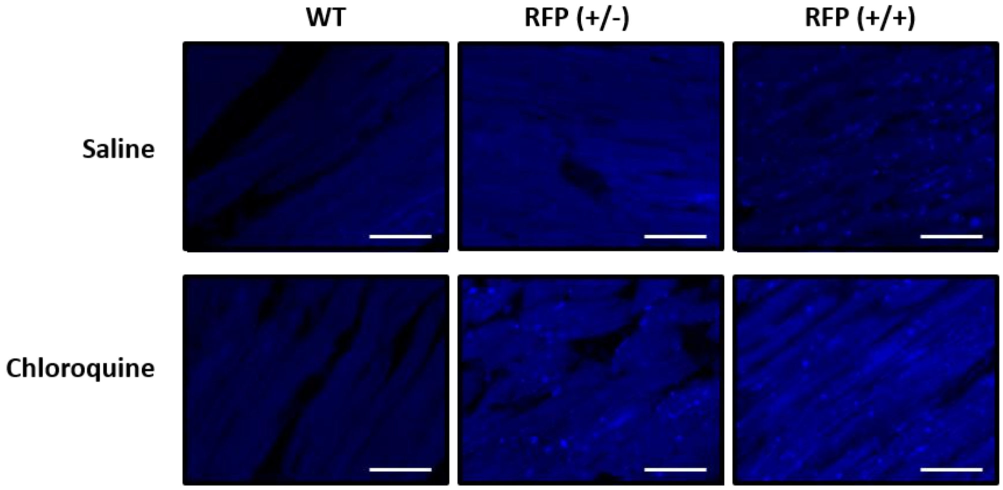

Fig S5. Chloroquine and monodansylcadaverine (MDC) injection

(A) 2 month-old wildtype and RFP+/+ heart were injected by chloroquine or saline for 4 h. The LV transverse sections stained by LC3B and p62 were shown. Increasing level and aggregated dots of LC3B and p62 were clearly seen in RFP+/+ cardiac myocytes, and at a reduced amount in RFP+/- myocytes (not shown). Chloroquine injection reduced p62 and LC3B level and the number of LC3B and p62 aggregates. Scale bar = 25 μm.

(B) Chloroquine and saline was injected as in (A), and MDC was injected 1 h before

sacrificing the animals. In RFP+/+ heart, many MDC-stained vacuoles were clearly visible. Chloroquine injection increased MDC fluorescence intensity in wildtype and RFP hearts, and increased the number of MDC-stained vacuoles in RFP+/- and RFP+/+ hearts, indicative of the blockage of lysosomal function by chloroquine. Scale bar = 25 μm.

(A)

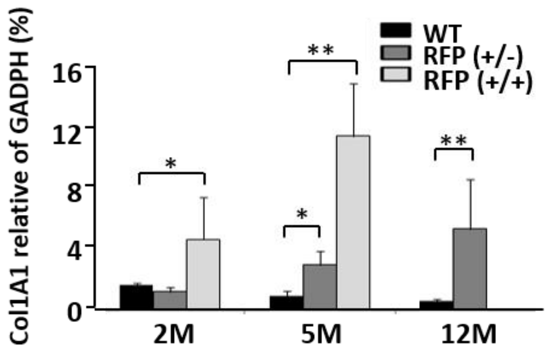

(B)

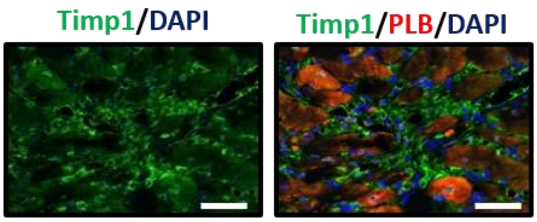

Fig S6. Collagen and TIMP1 expression in RFP mouse heart

(A) Col1A1 expression (normalized to GADPH) in LV samples of 2-, 5- and 12-month-old WT and RFP Tg mice. **, P < 0.01; *, P < 0.05 compared with the WT mouse. N=3 in each mouse group.

(B) TIMP1 expression in 2 month-old RFP+/+ mouse heart section. Left and right pictures showing the same section using TIMP1 (green), DAPI (blue), and phospholamban (PLB, red), indicating TIMP1 expression within cardiac myocytes (appearing as orange color) and at perivascular and interstitial regions. Scale bar = 50 μm.

Gene ontology of altered genes in RFP mouse heart

	RFP(+/-)			RFP(+/+)	
age	2M	5M	12M	2M	5M
Cardiotoxicity No of genes[a] (p-value)	Cardiac Damage 2 (10^{-1} -10^{-2})	Cardiac Necrosis /Cell Death 6 (10^{-2} -10^{-3})	Cardiac Dysfunction 11 (10^{-2} -10^{-5})	Cardiac Dysfunction 4 (10^{-1} -10^{-3})	Congenital Heart Anomaly 27 (10^{-1} -10^{-2})
	Cardiac Necrosis /Cell Death 6 (10^{-1} -10^{-2})	Cardiac Dysfunction 4 (10^{-2} -10^{-3})	Cardiac Hypertrophy 21 (10^{-1} -10^{-4})	Cardiac Inflammation 4 (10^{-1} -10^{-3})	Cardiac Inflammation 15 (10^{-1} -10^{-3})
	Cardiac Dysfunction 2 (10^{-1} -10^{-1})	Congenital Heart Anomaly 3 (10^{-1} -10^{-2})	Cardiac Infarction 10 (10^{-5} -10^{-5})	Cardiac Infarction 6 (10^{-4} -10^{-4})	Cardiac Infarction 17 (10^{-3} -10^{-3})
	Congenital Heart Anomaly 2 (10^{-1} -10^{-2})	Cardiac Inflammation 4 (10^{-1} -10^{-3})	Cardiac Fibrosis 13 (10^{-1} -10^{-4})	Pulmonary Hypertension 2 (10^{-2} -10^{-2})	Cardiac Dilation 18 (10^{-1} -10^{-2})
	Cardiac Hypertrophy 8 (10^{-1} -10^{-2})	Cardiac Infarction 4 (10^{-3} -10^{-3})	Cardiac Dilation 9 (10^{-1} -10^{-3})	Cardiac Fibrosis 5 (10^{-2} -10^{-3})	Heart Failure 17 (10^{-1} -10^{-2})

[a] Classified as being differentially expressed that relate to the specified function category; a gene may be present in more than one category

Table S1. Classification of genes differentially expressed in RFP mice heart

Top five categories of genes differentially expressed in RFP mouse hearts are associated with the cardiotoxicity phenotype. The number of differentially-expressed genes involved in a given phenotype (such as "cardiac hypertrophy" and "cardiac infarction") increases in accordance with the transgene dose (RFP zygosity), the age of mice, and the manifestation of the disease. N=3.
